# Challenges of Integrative Disease Modeling in Alzheimer's Disease

**DOI:** 10.3389/fmolb.2019.00158

**Published:** 2020-01-14

**Authors:** Sepehr Golriz Khatami, Christine Robinson, Colin Birkenbihl, Daniel Domingo-Fernández, Charles Tapley Hoyt, Martin Hofmann-Apitius

**Affiliations:** ^1^Department of Bioinformatics, Fraunhofer Institute for Algorithms and Scientific Computing, Sankt Augustin, Germany; ^2^Bonn-Aachen International Center for IT, Rheinische Friedrich-Wilhelms-Universität Bonn, Bonn, Germany

**Keywords:** Alzheimer's disease, challenges, integrative disease modeling, hypothetical, data-driven

## Abstract

Dementia-related diseases like Alzheimer's Disease (AD) have a tremendous social and economic cost. A deeper understanding of its underlying pathophysiologies may provide an opportunity for earlier detection and therapeutic intervention. Previous approaches for characterizing AD were targeted at single aspects of the disease. Yet, due to the complex nature of AD, the success of these approaches was limited. However, in recent years, advancements in integrative disease modeling, built on a wide range of AD biomarkers, have taken a global view on the disease, facilitating more comprehensive analysis and interpretation. Integrative AD models can be sorted in two primary types, namely hypothetical models and data-driven models. The latter group split into two subgroups: (i) Models that use traditional statistical methods such as linear models, (ii) Models that take advantage of more advanced artificial intelligence approaches such as machine learning. While many integrative AD models have been published over the last decade, their impact on clinical practice is limited. There exist major challenges in the course of integrative AD modeling, namely data missingness and censoring, imprecise human-involved priori knowledge, model reproducibility, dataset interoperability, dataset integration, and model interpretability. In this review, we highlight recent advancements and future possibilities of integrative modeling in the field of AD research, showcase and discuss the limitations and challenges involved, and finally, propose avenues to address several of these challenges.

## Introduction

Alzheimer's Disease (AD) manifests in a collection of symptoms including the deterioration of cognition, memory, and behavior which often leads to interference with activities of daily living. In 2017, AD ranked among the top five causes of death worldwide, with 2.44 million (4.5%) deaths from AD^1^^,^^2^. Worldwide, there are currently around 50 million people living with AD, and every 3 s a person develops this condition. It is estimated that only a quarter of those living with AD are diagnosed, and more than 17 million healthcare workers annually invest 18 billion hours of care, at a cost of more than one trillion US dollars to tackle AD-associated problems^3^^,^^4^. Extrapolating these statistics to the coming decades suggests the immense socioeconomic impact of AD on all involved parties: patients, caregivers, healthcare systems, and indirectly, the economy. Thus, strategies to reduce the global emotional and financial burden of AD are of great importance. To develop such strategies, a deeper understanding of the pathophysiology underlying AD is necessary and may lead to opportunities for earlier detection and therapeutic interventions.

In general, AD progression is categorized into three clinical disease stages: (i) During the pre-symptomatic phase, individuals may have already developed pathological changes that underlie AD, but remain cognitively normal, (ii) in the prodromal phase, often referred to as mild cognitive impairment (MCI), the first cognitive symptoms, commonly episodic memory deficits, appear. These symptoms can be acute, but they do not yet meet the criteria for dementia, (iii) in the dementia stage, impairments are severe enough to interfere with daily life (Jack et al., 2010).

Understanding of the etiology of AD is complicated due to the existence of dysregulations at different biological scales, ranging from genetic mutations to structural and functional alterations of the brain (Aisen et al., 2017). For this reason, significant efforts have been made in recent years to discover candidate markers for disease-related pathological changes throughout all modalities, including neuro-imaging, cerebrospinal fluid (CSF) samples and a broad variety of -omics data. Studies have successfully identified multiple biomarkers for neurodegeneration and AD (Blennow and Zetterberg, 2018). However, effectively translating extensive biomarker screenings into clinical application remains a challenging task, because individual biomarkers can only provide a highly incomplete view on such a multifactorial disease (Younesi and Hofmann-Apitius, 2013). For instance, while multiple associations between genetic variants and AD have been established (Jansen et al., 2019; Kunkle et al., 2019), none of these associations fully describe disease pathogenesis. As a result, one of the major challenges in AD research is translating diverse biomarker signals available into multimodal, multiscale models of disease pathogenesis.

In recent years, a new translational research paradigm called “integrative disease modeling” has emerged, to address this challenge (Younesi and Hofmann-Apitius, 2013). It aims at modeling heterogeneous measurements across different biological scales, in order to provide a holistic picture of biomarker intercorrelations in the disease of study. To this end, advanced high-throughput technologies and neuroimaging procedures are being used to collect data from multiple modalities. These diverse data need to be integrated, that is, combined in a way that preserves the structure and meaning in the data, using computational algorithms. Only then can they provide a solid basis for further analysis such as reasoning, simulation, and visualization. In order to contribute to understanding of the complex pathophysiology of the disease, the results should be actionable and thus must be interpretable. Integrative disease modeling, by collecting, integrating, analyzing, and ultimately interpreting the measurements, facilitates the understanding of the pathophysiology of complex diseases like AD (Hampel et al., 2017).

Existing integrative models in the context of AD can be placed in two primary categories, namely hypothetical models and data-driven models (Table 1). Hypothetical models are non-numerical and rely on reasoning over findings of previously published studies (Jack et al., 2010), rather than large amounts of data. By including this prior knowledge, these models try to detail the temporal changes of AD biomarkers relative to each other as well as to clinical disease stages and trial endpoints.

**Table 1:** Organization of and references for data-driven integrative AD models.

**Data-driven integrative AD models**			**References**
Traditional			Caroli and Frisoni, 2010; Jack et al., 2011, 2012
Machine learning	Generative		Fonteijn et al., 2012; Chen et al., 2016; Khanna et al., 2018; Oxtoby et al., 2018; Basu et al., 2019; De Jong et al., 2019; Gootjes-Dreesbach et al., 2019; Martinez-Murcia et al., 2019
	Discriminative	Supervised	Hinrichs et al., 2010; Magnin et al., 2010; Rao et al., 2011; Zhang et al., 2011; Da et al., 2013; Li et al., 2013
		Unsupervised	Nettiksimmons et al., 2014; Gamberger et al., 2017; Toschi et al., 2019

*We subdivide data-driven integrative AD models which into two subgroups. While the first group uses simple statistical approaches (e.g., simple linear models), the second group uses more advanced techniques (e.g., machine learning). The advanced machine learning models include generative and discriminative models, the latter of which can be classified as either supervised or unsupervised models*.

By contrast, data-driven integrative models take advantage of developments in computational approaches and big data. For the sake of this review, we will distinguish between two subcategories of data-driven models. The first covers traditional statistical methods of generally lower complexity, such as linear models. Often, these models are used to estimate biomarker trajectories by regressing measured data against a prespecified dependent variable, such as a clinical readout or the disease stage (Bateman et al., 2012). The second subtype exploits more advanced artificial intelligence approaches such as machine learning. Within this subtype, models can be characterized as discriminative or generative. Discriminative models are designed to discriminate between groups (e.g., cases and controls) and can be further described as supervised or unsupervised, depending on whether they rely on labeled (Hinrichs et al., 2011; Da et al., 2013) or unlabeled (Toschi et al., 2019) data. Generative models contribute to disease understanding by automatically learning the inherent distribution of a dataset and its feature interdependencies (Oxtoby et al., 2018). An exemplary application is the extraction of disease progression signatures as demonstrated by the ensemble of Bayesian networks developed by Khanna et al. (2018).

Integrative AD modeling faces many challenges. Hypothetical models, by their nature, are time-intensive to construct and require specialist knowledge. Their primary role in AD research is to provide ideas for future experiments. Likewise in data-driven modeling, several challenges at each step of the process (i.e., collection, integration, analysis, and interpretation) must be addressed. Data missingness and data censoring are significant bottlenecks in data collection as well as analysis and interpretation. Meanwhile, the heterogeneity and complexity of biological data are major impediments to data integration, which forms the basis for all data-driven approaches. Furthermore, data mapping, data labels, and biased data are additional barriers to robust data analysis and interpretation. Finally, insufficient numbers of subjects restrict the statistical power of data-driven integrative AD models. These fundamental challenges explain why, at this point in time, although many integrative AD models have been published over the last decade, their impact on clinical practice is limited.

In this review, we highlight recent advancements and future possibilities of integrative modeling, discuss the limitations and challenges involved, and finally, propose avenues to address several of these challenges, in the context of AD research.

## Integrative AD Models

As already mentioned, integrative AD models can be characterized as either hypothetical or data-driven, each of which has strengths and weaknesses. In the following, we compare different models of each type and discuss their benefits and limitations. Finally, we elaborate on how associated limitations and challenges could be handled.

### Hypothetical Models

In hypothetical modeling, a model is generated about an object of study, direct knowledge of which is difficult to obtain. These models provide hypotheses about the object (Gladun, 1997). In integrative AD modeling, researchers develop so-called cascade models, in which the measurements of a set of biomarkers are normalized and their trajectories are plotted on a common time scale, aligned to disease stages (Jack et al., 2010, 2013). These models are typically developed by reviewing the available knowledge and reasoning over observations from previously published studies. They are not directly informed by measured data.

One of the first hypothetical integrative AD models was developed by Jack et al. (2013) [revised from a previous model (Jack et al., 2010)]. This model hypothesized the temporal changes of the five most studied biomarkers of AD pathology in relation to estimated years from expected symptom onset and in relation to other biomarkers. These biomarkers are CSF amyloid-beta protein (CSF Aβ_1−42_) and tau protein (CSF tau) levels, amyloid-beta PET imaging (PET Aβ), Fluorodeoxyglucose-PET imaging, and structural MRI readouts. In this cascade model, the authors presumed that biomarker trajectories should exhibit a sigmoid-shaped curve. This imposition is a direct result of the limited sensitivity of measurements at time extremes, which the authors addressed by taking the floor of the measurements at early timepoints, and the ceiling of the measurements at late timepoints. The authors hypothesized that the two amyloid-beta (Aβ) biomarkers (i.e., CSF Aβ_1−42_ and PET Aβ imaging) gradually approach an abnormal state while the subject remains in a cognitively normal state. After a lag period, the length of which varies from patient to patient, and in later disease stages, CSF tau, Fluorodeoxyglucose-PET, and structural MRI biomarkers follow the same pattern and begin the transition to an abnormal state. Similarly, Frisoni et al. (2010) established a theoretical progression of cognitive and biological markers (primarily imaging features) based not only on the clinical disease stages, but also patient age at AD diagnosis and time since diagnosis. Although both models captured earliest detectable changes in amyloid markers, Frisoni et al. (2010) additionally theorized that these changes plateau by the MCI stage, when the individuals are no longer cognitively normal. Furthermore, they suggested that F-fluorodeoxyglucose PET is abnormal by the MCI stage and continues to change well into the dementia stage. Structural changes appear later, following a temporal pattern mirroring tau pathology deposition, which slightly differs from the Jack et al. models (Jack et al., 2010, 2013).

While hypothetical models cannot be directly applied, they can be used to suggest directions for future experiments that themselves would address diagnosis, prediction, or decision making tasks (Gladun, 1997). However, there are a number of challenges relating to the construction of hypothetical models. In the following, we discuss these challenges and propose ways to address some of them.

### Challenges of Hypothetical Models

The exclusive reliance of hypothetical models on literature presents several challenges. First, relevant literature must be identified. Second, the scientific knowledge contained in the literature must be extracted in a meaningful form. Finally, the knowledge has to be modeled.

In order to build a hypothetical model, a researcher must identify a set of relevant publications, called a literature corpus, which accurately reflects AD knowledge. This corpus should be representative of the relevant aspects of AD, contain the most up-to-date publications, and not be biased toward subfields or trends. However, the number of new AD publications has increased each year since 2005, and there were nearly 15,000 such publications in 2017 alone (Dong et al., 2019). With such publication rates, it is challenging for researchers to manually create high quality corpora (Rodriguez-Esteban, 2015), Moreover, manual generation of these corpora is susceptible to bias, because researchers may tend to draw more heavily from authors or subfields with which they are more familiar (Atkins et al., 1992). The size of a corpus will also be limited by the time and resources available to the researchers. However, text mining has been used effectively to automatically classify relevant literature, based on titles and abstracts (e.g., see Simon et al., 2018), and to prioritize texts (Singh et al., 2015). Publications identified by this classification can be directly taken as the corpus or used as a more manageable set of publications from which the domain experts can appropriately select. Hypothetical models are susceptible to biases present in the literature (Boutron and Ravaud, 2018), but a well-designed, computationally selected corpus can mitigate the effects of those biases.

Once the corpus has been identified, the challenge of knowledge extraction remains. The goal here is to recover the knowledge contained in the publications in a meaningful way. Conducting this task manually is a time-consuming process that requires a high degree of domain knowledge. Here, text mining poses the opportunity to extract knowledge in a computable form (Gyori et al., 2017; Lamurias and Couto, 2019). Moreover, it significantly reduces the amount of time required to read publications, which enables significantly larger corpora to be used in the building of hypothetical models.

Finally, in order to build hypothetical models, the information gleaned from the literature corpus must be organized in a coherent way. The entities and the relationships between them should all be represented. Mind maps provide a non-automated way of generating a knowledge model, driven by domain-expert knowledge (Kudelic et al., 2011). However, if automated information extraction strategies were used on the literature corpus, then knowledge graphs are well-suited for storing the extracted knowledge (Gyori et al., 2017). A major advantage of this strategy is that the knowledge graph is computable, meaning downstream machine learning tasks can be carried out for knowledge discovery. Furthermore, knowledge graphs support hypothesis generation by enabling researchers to assess whether their hypotheses are compatible with existing knowledge (Humayun et al., 2019).

Automated methods of corpus identification, knowledge extraction, and knowledge modeling provide a means of mitigating the challenges of hypothetical modeling. They reduce the time burden, mitigate the risk of bias in manual methods, and generate computable knowledge representations. This can yield more reliable hypothetical AD models.

Hypothetical models are non-numerical and rely exclusively on qualitative information, gleaned from a review of previous findings. This limits their usability solely to eliciting hypotheses for future experiments. They are neither predictive nor can they be used for analysis of any kind of data. They are meant to represent a kind of “typical” AD progression, without reflecting individual deviations from that. Given the broad biological heterogeneity observed among AD subjects, and the increasing relevance of personalized medicine (Reitz, 2016), there is a need for models that are capable of achieving this.

Data-driven models built on data collected in longitudinal cohort studies can serve to support or challenge hypotheses generated by hypothetical models (Petrella et al., 2019). Data-driven models are appropriate for a wide range of tasks that lie beyond the scope of what hypothetical models are designed for. For example, using data models can capture individual subject particularities that hypothetical models cannot (see e.g., Young et al., 2015). In the following, we discuss data-driven models and their challenges in depth.

### Data-Driven Models

In contrast to hypothetical models, data-driven integrative models are directly derived from datasets comprising readouts of multiple biomarkers. Such models can be applied to a broad variety of tasks ranging from predictive modeling e.g., predicting patient diagnosis (Ding et al., 2018) or age at disease onset (Chuang et al., 2016; Peng et al., 2016) to discovering patterns in the data that shed light on biomarker interdependencies and disease underlying mechanisms. Since these models use extensive data, they are not limited by preconceived notions in the way that hypothetical integrative models are.

Data-driven AD models can be classified into two primary subtypes based on the statistical approaches and algorithms applied (Table 1). The first subtype use traditional statistical methods such as linear modeling, and the second employs artificial intelligence and more specifically machine learning approaches.

#### Traditional Statistical Models

In AD modeling, traditional statistical approaches, such as linear mixed-effects models, are often used to estimate biomarker trajectories (Caroli and Frisoni, 2010; Jack et al., 2011, 2012). In these models, measured data, are regressed against a prespecified variable, such as disease stage, to detail the temporal changes of AD biomarkers during the course of disease. Essentially, these models provide empirical testing of hypothetical multiple biomarker trajectory plots.

Jack et al. (2012) used linear mixed-effects models to investigate the shape of five important AD biomarker trajectories (i.e., Aβ_42_, tau, amyloid, fluorodeoxyglucose PET, and structural MRI) as a function of a cognitive test score, the Mini-Mental State Exam (MMSE). This model parameterization enabled them to assess within-subject rates of biomarker changes with respect to changes of the MMSE score. They found that lower baseline MMSE scores are correlated with worse baseline biomarker values and that higher rates of biomarker change were associated with worsening MMSE score. This model constructed the biomarker trajectories without making any assumptions about the shapes of the trajectories. This contrasts with the authors' earlier hypothetical biomarker cascade model, which imposed a sigmoid trajectory curve.

While the shapes of the trajectories in this data-driven model agree with the assumptions made in the hypothetical exemplar, the model has several limitations, pertaining to model design choices and deficiencies in the data. The authors chose to use the MMSE score as the independent variable. This choice was made because the MMSE score provides a linear measure of disease progression that was available across all datasets. However, this introduces challenges in the estimation of trajectories in early disease stages, because MMSE scores in cognitively normal patients are relatively stable over time (Tombaugh, 2005), yielding only a narrow range of values. Moreover, especially when studying early disease stages, the model additionally suffers from possible absence of information on future disease developments of a subject. This absence of data on future disease outcome is related to data censoring, which will be addressed in more detail later.

In their data-driven model (Jack et al., 2011), Jack et al. aimed to unravel the temporal order of biomarker trajectories becoming abnormal, rather than only describing the shape of their trajectories. They used the prevalence of biomarker abnormalities at different disease stages to empirically assess the temporal ordering of their trajectories. They employed generalized estimating equations, a generalized linear model for longitudinal data that can deal with correlated observations, to evaluate and compare the proportion of abnormal observations per biomarker. The proper choice of a cut-off defining when biomarker measures are considered to be abnormal is a point of debate and making this choice requires critical judgement. To differentiate between normal and abnormal biomarkers, Jack et al. (2011) determined a cut-off by looking at an independent post-mortem cohort. However, since, by construction, results were highly sensitive to the selected cut-off for each biomarker, the temporal resolution of the model is limited.

While the proportion of patients with abnormal biomarker values might seem an unnatural choice for comparing biomarkers, alternative strategies also have drawbacks. Caroli and Frisoni (2010) computed Z-scores based on values of each biomarker and fitted them against Alzheimer's Disease Assessment Scale-Cognitive Subscale (ADAS-cog) scores, comparing linear and sigmoidal fits. Their investigation showed that a sigmoid curve fit the observed data significantly better than a linear one for most of the biomarkers, and thereby might be able to characterize the time course of those biomarkers. These results were consistent with the hypothetical model proposed by Jack et al. (2010) and Jack et al. (2013). However, the biomarker trajectories cannot be directly compared with the data-driven model developed by Jack et al. (2011), since different scales were employed in both studies. While standardization of values by converting them into Z-scores resolves this problem, it introduces a new one: by definition, the arithmetic mean of each biomarker will be 0. This makes it impossible to reasonably compare biomarker distributions based on their means using standard statistical procedures like, for example, *t*-tests (Jack et al., 2011; Moeller, 2015).

The arbitrariness of defining a cut-off for abnormality of a biomarker will always pose a limitation on statistical approaches relying on biomarkers. While such cut-offs simplify the interpretation of the biomarker, there is no universally correct cut-off for a given biomarker. Rather, appropriate cut-offs heavily depend on the population, and even the individual, on which a biomarker will be used. Covariates such as an individual's age, genetic risk factors, and family history of AD must be considered. For these reasons, there is no single optimal cut-off for any given biomarker (Bartlett et al., 2012; Anne and Fagan, 2014). To address this, a less rigid technique has been developed, that designates an intermediate range using two cut-offs, one permissive and the other conservative (Klunk et al., 2012; Jack et al., 2016a,b; Bzdok, 2017). The permissive point can be used for earliest detectable evidence of AD pathologic changes and the conservative one for high diagnostic certainty. Moreover, different statistical approaches, like Youden's index and the receiver operating characteristic (ROC) curve, can be applied to help determine an appropriate cut-off.

Linear traditional models are ill-equipped to handle the increasingly high-dimensional data being collected in AD studies. Thanks to recent technological advancements, the granularity of AD datasets with respect to information resolution, feature size, and complexity of meta-information have increased. For example, improved neuro-imaging techniques generate datasets with higher resolution than previously available. This information distributed over voxels, a 3D imaging unit, is hard to capture using linear models (Bzdok, 2017). Therefore, more advanced data-driven models have been developed based on machine learning. These models are generally more flexible and compatible with the complex datasets encountered in biology research (Bzdok, 2017).

#### Machine Learning Models

Machine learning models can be characterized as generative or discriminative. As previously mentioned, discriminative models are designed to differentiate between groups, while generative models provide better disease understanding by learning inherent properties from datasets, such as feature interdependencies.

##### Generative models

Generative modeling relies on the use of statistics and probability to extract patterns from data and learn the underlying distribution. In the following, three types of generative integrative AD models are reviewed: event-based models, Bayesian network learning, and autoencoders.

*Event-based models.* Event-based models estimate the most probable sequence of events based on the assessment of a probability density function for a particular event order. Fonteijn et al. (2012), Chen et al. (2016), and Oxtoby et al. (2018), used this method to learn the sequence of AD events based on imaging and non-imaging measurements from a clinical study. The authors first fitted simple mixture models (e.g., gaussian mixture models) to individual biomarkers in order to calculate the likelihood of the normality or abnormality status per biomarker. Given these likelihoods, by multiplication of the probabilities, the likelihoods for each possible order of events was calculated. The order with the highest probability was then selected using a greedy Markov Chain Monte Carlo algorithm to describe the temporal correlation of the biomarker trajectories over the course of AD progression.

The models developed by Fonteijn et al. (2012) and Chen et al. (2016) simplified the sequence of biomarker abnormalities over the course of the disease progression by relying on the assumption that all subjects follow a single event sequence. However, AD is highly heterogeneous and includes distinct subgroups (Ferreira et al., 2018). To account for this, Young et al. (2015) established their event-based models with two extensions: a Mallows model and a Dirichlet process mixture of generalized Mallows models. The first extension allows subjects to deviate from the main event sequence, and the latter clusters subjects according to different event sequences.

In principle, the event sequence proposed in the hypothetical model is similar to that observed using traditional and event-based models. Changes in CSF measures are the earliest events, followed by regional brain atrophies and finally succeeded by diminished cognitive scores. However, the event sequence in the hypothetical and traditional models is constructed based on predefined clinical assessments and often imprecise or subjective cut-offs. By contrast, in generative models, the sequence of events, as well as the clustering of biomarkers into normal and abnormal classes, is directly extracted from the data (e.g., the onset of a new symptom, like memory performance decline). Thus, event-based models explain the changes without a priori biases. Moreover, generative models are able to characterize uncertainty in the event ordering arising from heterogeneity in the population and thus, can address individual deviations from the generic model.

*Bayesian network learning.* Extensive research efforts have been made to uncover the relationships between individual biomarkers and AD. Yet the number of studies that investigated the interplay between multiple biomarkers themselves is comparably limited. Khanna et al. (2018) and Ding et al. (2018) built Bayesian network models covering different biological scales and time points to uncover the interplay amongst sets of biomarkers. Ding et al. (2018) considered the ApoE allele, PET and MRI imaging data, scores from psychological and functional tests, and the medical history of patients with respect to neurological diseases. Using a variety of feature selection metrics, they determined the most relevant features with respect to the clinical dementia rating and modeled these heterogeneous measurements using a Bayesian network to determine their probabilistic interdependencies. However, these models only capture conditional probabilities between predictor variables and clinical outcomes. They are unable to provide a causal mechanistic understanding of an observed phenomenon. Such hypothesized pathophysiological mechanisms are important for making reliable predictions and having confidence in the practical application of data-driven models. To this end, Khanna et al. (2018) employed a combination of data-driven probabilistic and knowledge-driven mechanistic approaches. They modeled clinical variables, genetic variants, pathways, and neuro-imaging readouts using Bayesian network learning to estimate dependencies between disease relevant features. Together with a cause-and-effect knowledge model derived from scientific literature, they partially reconstructed biological mechanisms that could play a role in the conversion of normal/MCI into AD pathology.

*Autoencoders.* The last type of generative model discussed in this review is autoencoders. In essence, an autoencoder is a neural network that aims to encode the input data into a lower dimensional representation and from that decode it again, reconstructing the original input. It has successfully been applied for different tasks on AD cohorts (Basu et al., 2019; Martinez-Murcia et al., 2019). The two main applications of this approach in the field consist of classifying patients based on AD diagnosis (Basu et al., 2019) and clustering of patient trajectories into subgroups (De Jong et al., 2019). These strategies are especially interesting for patient classification and stratification tasks in datasets where information is sparse. However, another novel and promising task for autoencoders is the generation of synthetic data from real patient level data (Gootjes-Dreesbach et al., 2019). This, in turn, could be used to circumvent legal and ethical constraints that restrict data sharing.

##### Discriminative models

Discriminative models are a class of models generally used for classification. Discriminative models that rely on labeled data are called supervised models, while unsupervised models use unlabeled data.

*Supervised discriminative models.* Diverse supervised discriminative methods such as support vector machines (SVM; Magnin et al., 2010), and multiple-kernel SVM (MKL; Hinrichs et al., 2010; Zhang et al., 2011) have been used to classify AD patients, MCI subjects, and controls. However, studies that used multiple-kernel SVM reported superior classification performance, because the use of multiple kernels facilitates the integration of multimodal biomarker data (Zhang et al., 2011). Additionally, MKL are well-suited for dealing with very high dimensional data (Young et al., 2013). MKL also enable individual weighting of biomarker modalities. This offers more flexibility for kernel combination and thus, a better integration of the data. For example Hinrichs et al. (2010), applied MKL in combination with MRI and PET imaging to differentiate between AD subjects and controls. Their method showed high classification performance, achieving 92.4% accuracy. Similarly, Zhang et al. (2018) combined MRI, PET, and CSF biomarkers to discriminate between healthy controls and AD/MCI. After integrating all biomarker data using a MKL, they deployed a linear SVM for the actual classification task, which resulted in 93.2% accuracy for classifying AD and healthy controls and 76.4% for discriminating between MCI and healthy controls. Both studies applied a similar method for classification, yet the latter one achieved a slightly higher accuracy. Comparing the approaches applied in Zhang et al. (2018) and Hinrichs et al. (2010) it becomes clear that the major reason for the difference in performance is the feature selection process. Depending on the available sample size, other methods might prove more promising (Liu et al., 2012). Moreover, Zhang et al. (2018) benefits from employing three biomarker modalities, namely, CSF measurements and two imaging modalities, compared to Hinrichs et al. (2010) who only use the two imaging modalities.

While the above kernel-based pattern recognition approaches yield categorical class decisions, Young et al. (2013) used gaussian process classification, which is a probabilistic classification algorithm. This study integrated imaging, CSF, neuropsychological, and genetic biomarkers to classify MCI subjects who remained stable and MCI patients who converted to AD within 3 years. In contrast to MKL, the probabilistic classification afforded by the gaussian process approach provides the opportunity to position the subjects according to disease stage, to stratify patients, and to model the sequence order of biomarker abnormality.

Another type of discriminative model is disease risk models. This type of supervised model can be used to predict the time to AD diagnosis for normal/MCI patients. Multiple approaches have been used to develop risk models for AD (Da et al., 2013; Li et al., 2013). Li et al. (2013) used a combination of cox regression analyses and time-dependent ROC approaches to evaluate prognostic utility and performance stability of candidate biomarkers. The authors deduced that both baseline volumetric MRI and cognitive measures can predict progression from MCI to AD. However, in participants' follow-up visits, only cognitive measurements remained predictive. Da et al. (2013) employed the cox proportional hazards models to compare the magnitudes of the relative association between predictors (patterns of brain atrophy, cognitive assessments, genetics, and CSF biomarkers) and time to conversion from MCI to AD. They concluded that brain atrophy and cognitive assessments in combination offer the highest predictive power of conversion from MCI to AD.

Although the results in both studies were similar, the time-dependent ROC curve used by Li et al. (2013) enabled them to predict disease risk as a function of time. Thus, this method provides clear benefit for a progressive disease such as AD, in which both the disease status and biomarker measurements change over time (Kamarudin et al., 2017).

The data labeling which enables supervised discriminative models to determine decision boundaries for distinguishing classes of interest can also introduce errors. Inaccurate labels will negatively affect the performance of the classifier. Such mislabeling is not uncommon in AD, due to the absence of a clear diagnostic biomarker (Fischer et al., 2017). Instead, diagnosis is currently made based on symptoms (Schott and Petersen, 2015) Furthermore, integrative data analysis is further complicated by the fact that the diagnostic criteria for MCI have changed over the years, and MCI is not consistently defined across clinical studies. While one study relies on assessing only a single cognitive domain for MCI diagnosis, such as speech or memory, others base their diagnoses on performance on cognitive tests for multiple domains. Apart from that, there are multiple pathologies for MCI; AD is just one of them. Thus, unified clear disease definitions are crucial, since the MCI classification accuracy can influence outcomes of research and clinical practice (Jak et al., 2010).

*Unsupervised Discriminative Models.* Unsupervised discriminative models use a variety of clustering techniques on unlabeled data, avoiding the challenges of data label accuracy. These techniques use properties of each data point to iteratively form groups, called clusters. This ultimately leads to a discrimination of the data into several clusters of highly similar data points. Given the observed biological heterogeneity among normal control subjects, Nettiksimmons et al. (2014) hypothesized that different subgroups may also be found among the MCI subjects. Using agglomerative hierarchical clustering, they sorted subjects based on MRI volumes, CSF measurements, and cognitive tests. Next, the resulting clusters were explored with regard to longitudinal atrophy, conversion time, and cognitive trajectories. Four clusters with unique biomarker patterns resulted: (i) a cluster biologically similar to normal controls. MCI patients from that cluster rarely converted to AD, (ii) one cluster with early AD pathology characteristics, (iii) another cluster of subjects with hardly any tau abnormality, but a high proportion of AD converters, and (iv) and finally one cluster with pre-AD symptoms wherein almost all subjects converted to AD. Based on these findings, they hypothesized that clusters ii and iv reflected the amyloid cascade pattern (Ricciarelli and Fedele, 2017) since both clusters presented lower CSF Aβ levels and elevated tau proteins. However, the tau level in cluster iv was higher, and more severe atrophy as well as cognitive impairment were detected. The authors concluded that more tau accumulation may lead to more cognitive decline. One of the intrinsic limitations of their clustering approach is that the number of clusters must be predefined. The maximum gap statistic is one approach to determine this number (Tibshirani et al., 2001). However, specifying the number of clusters beforehand will always bias the clustering to some extent, and choosing a reasonable number is no trivial task given the broad variety of subtypes found among AD subjects.

Toschi et al. (2019) used Density-Based Spatial Clustering of Applications with Noise (DBSCAN; Thanh et al., 2013), which does not require pre-specifying the number of clusters. They integrated five validated CSF biomarkers in order to cluster a cohort where symptomatic patients presented diagnoses ranging from self-perceived cognitive decline (Zhang et al., 2011) to MCI to AD. In contrast to the previous study, Toschi et al. (2019) adjusted all biomarker values for age, sex and their interactions to exclude them as confounders (Pourhoseingholi et al., 2012). Moreover, Toschi et al. (2019) used t-Distributed Stochastic Neighbor Embedding (t-SNE) to reduce the dimensionality of biomarkers space, since defining the distance between the data points in a high dimensional space of biomarkers is notoriously difficult (Domingos, 2012). Finally, they applied DBSCAN on this lower dimensional representation. DBSCAN defines a high data density region based on two parameters: (i) the radius of the neighborhood, and (ii) the minimum number of points within the radius. These values are determined by a nearest neighbor method, in which the distance of each point to their nearest n points is calculated. Afterwards, results are sorted, plotted and the value with most pronounced change is selected as the optimal value. Using DBSCAN, Toschi et al. (2019) characterized five biological clusters which were not significantly bound to the original distinct clinically phenotyped diagnostic groups. They explained that the clusters included all phenotypic groups and were not homogeneous enough to be considered as a specific AD pathophysiology. Moreover, contrary to general belief that Aβ_1−42_ is linearly associated with the progression of AD and cognitive decline (Sperling et al., 2011a; Samtani et al., 2013), their findings suggest that Aβ_1−42_ is less likely to contribute to phenotypic discrimination.

The dimensionality reduction technique, t-SNE, used by Toschi et al. (2019) enabled them to better separate the data and hence, to enhance cluster identification, in comparison to directly running a clustering algorithm on a high dimensional data as Nettiksimmons et al. (2014). However, their main limitation is that clustering results are highly sensitive to two parameters necessary for DBSCAN. Moreover, they did not include other biomarkers, such as imaging and genetics biomarkers, which could enhance their clustering, as previously reported by Young et al. (2013, 2018).

Unsupervised clustering algorithms are ideal for identifying subgroups and non-linear associations between individuals based on a multidimensional profile, regardless of the individual labels, in contrast to supervised algorithms. This allows the grouping of individuals based on shared pathophysiological drivers and triggers and, possibly, similar longitudinal disease trajectories. This is an advantage in the AD field due to the prevalence of unreliable labels stemming from misdiagnosis and to the biological heterogeneity of AD subjects. On the other hand, most unsupervised clustering algorithms perform better with a larger sample size than is often obtainable in AD studies (Oxtoby and Alexander, 2017). Therefore, the smaller size inherent to AD cohorts may lead to clustering instability.

To this point, we have reviewed a broad variety of data-driven integrative AD models and elaborated on their associated limitations and challenges. In the following, we enumerate more general challenges researchers encounter in the course of data-driven integrative AD modeling and suggest how these could be addressed.

### Challenges of Data-Driven Modeling

Although there exists a wide range of data-driven integrative modeling approaches, not all of them are well-suited for every analytic task and each has its own strengths and weaknesses. Still, there are some challenges which affect all data-driven approaches to some degree: data collection, reproducibility of findings, and interpretability of models and results.

#### Data Collection

Collecting patient level data, the basis for all data-driven modeling, is a time-consuming and costly process. Additionally, it is a source of major challenges and limitations of these models. In particular, data “censoring” and “missingness,” can impede modeling, bias models, or even make certain modeling techniques unfeasible.

Data censoring describes the condition in which a particular event (here AD diagnosis) is not observed for certain study participants during the study runtime. This censoring can occur in two ways: if AD diagnosis occurred before the start of the study; or if the patient drops out of the study, or the study ends without occurrence of the AD diagnosis event. A significant number of patients enrolled in clinical studies have already received a diagnosis before the beginning of the study, indicating that they are in a progressed stage of the disease (Ellis et al., 2009). It is therefore not possible to obtain indications of early disease onset in such patients. The second form of censoring arises from two sources. First, all observational cohort studies experience participant dropout for a variety of reasons, including the participation burden on caregivers or medical problems (Coley et al., 2008). Second, subjects that remain healthy throughout study runtime could still develop the disease after the study ended, meaning they were in a prodromal disease stage. It is thus impossible to know if or when the patient would eventually receive an AD diagnosis. This form of censoring is common in longitudinal AD studies, because AD is a slow-progressing disease, while the studies are typically quite short (Lawrence et al., 2017), due to limited funding (Prabhakaran and Bakshi, 2018).

Disease onset is a critical point for clinical intervention (Sperling et al., 2011b), so it is subject to extensive research efforts. It is here, however, where data censoring impedes data analysis the most. Data censoring can result in over- or under-sampling of early and advanced disease stages. This, in turn, leads to models biased toward specific disease stages (Ning et al., 2010). Various methods, such as complete data analysis (Xiang et al., 2013), imputation (Fisher et al., 2019), or analysis based on dichotomized data (Donohue et al., 2011), have been established to address censored data. Yet all of these methods may introduce error and impose complexities and biases on other integrative modeling steps, such as model interpretation, and thus need to be used with care (Prinja et al., 2010).

The complete absence of a value for variables in the observation of interest likewise poses a significant challenge to data-driven modeling. This missing data in AD cohort studies occurs for several reasons, including unwillingness of patients to undergo invasive tests like lumbar punctures, and the high cost of measuring a particular variable, such as imaging scans (Engelborghs et al., 2017). The implications of such a scenario include a loss of statistical power of the study and may bias the conclusions that can be drawn (Hughes et al., 2019). Over the past decades, novel statistical methods (Molenberghs et al., 2014) and software (Quartagno and Carpenter, 2016; Moreno-Betancur et al., 2017) have been developed for analyzing data with missing values. However, analysis restricted to individuals with complete data is generally preferred, if feasible.

Despite the challenges in collecting complete and uncensored data, the value of data in strengthening disease understanding is clear. Several large-scale AD patient datasets have been collected for use in a variety of studies (Lawrence et al., 2017) including, for example, Alzheimer's Disease Neuroimaging Initiative (ADNI; Mueller et al., 2005), Australian Imaging Biomarkers and Lifestyle Study of Aging (AIBL; Ellis et al., 2009), the Dominantly Inherited Alzheimer Network (DIAN; Moulder et al., 2013), and European Prevention of Alzheimer's Dementia (EPAD; Vermunt et al., 2018). However, these classical observational studies are subject to bias, resulting from the inclusion and exclusion criteria used to select participants (Miksad and Abernethy, 2018).

The use of electronic medical records (EMRs) has been proposed as a potential solution to reduce the bias of classical clinical trials. They provide an alternative view on patient measurements (Fröhlich et al., 2018), so, a collection of EMRs can provide a more representative view on patient measurements. However, EMRs are largely phenotypic: molecular phenomena such as genomic variants are not reflected in the data. Moreover, extracting information from EMRs requires natural language preprocessing, which itself currently remains a difficult and error-prone process.

#### Reproducibility

The ability to reproduce the findings of a study using different subjects is an important part of scientific research. This is particularly the case in integrative AD modeling, since the tendency of AD datasets is not to fully reflect the diversity of AD patients. Inclusion-exclusion criteria in clinical studies can lead to significant under-representation of some populations. For example, the landscape of data-driven AD models is currently dominated by only a few cohorts which are made up largely of White Caucasians, and, to a lesser extent, are constrained by geographic location (Lawrence et al., 2017). Since most observational cohorts are not representative of the general AD population (Ferreira et al., 2017), it is important to validate the resulting models with an independent cohort study. While this external validation is a necessary step to corroborate findings, it is complicated by data interoperability and sample size.

##### Interoperability

The ability to map the data coming from one study to data from another study is known as data interoperability^5^. Each of the major AD clinical studies was established with a specific sample and feature characterization. Since they might not be directly interoperable, extensive curation is needed before the external validation of a model can be carried out. Otherwise, the training cohort and the validation cohort would be based on different populations, and would contain different measurements. Thus, before validation, researchers must map and assess the “comparability” of both features and subjects.

Feature mapping requires specifying relationships between data elements from different data models and standardizing the terms used to represent the features in the two datasets. This is due to the fact that controlled vocabularies are not used to annotate the datasets. Thus, even if the same biomarker has been collected in two studies, it is usually referred to by different terms, impeding a direct comparison of the datasets. For example, the hippocampus is one of the earliest sites of AD pathology, and hippocampal volume is measured in ADNI and EPAD. However, ADNI identifies this biomarker as “Hippocampus,” while EPAD refers to it as “lhvr” (right hemisphere) and “lhvl” (left hemisphere).

Moreover, the subject populations in each study must be comparable. For instance, if the biological sex distributions in two AD studies differ significantly, then the cognitive impairment scores of the cohorts cannot be directly compared, because female AD patients have been shown to have greater cognitive impairment than men in comparable stages of the disease (Laws et al., 2016).

There are several strategies to overcome the lack of interoperability between datasets at both feature and subject level. At the feature level, interoperability can be attained by annotating datasets according to a standard controlled vocabulary. Several such vocabularies (e.g., NIFT Iyappan et al., 2017 and PTS Iyappan et al., 2016) have been established, but significant improvements in interoperability will only come with widespread adoption (Neu et al., 2012). The most prominent example might be the AD specific standard developed by the Clinical Data Interchange Standards Consortium (CDISC; Neville et al., 2017). At the subject level, mapping between training and validation cohorts can be accomplished by identifying, in the validation cohort, a subset of subjects that is statistically comparable to the training cohort. Finally, in order to assess the comparability of subjects from different studies, techniques such as statistical matching can be used (Austin, 2011).

##### Sample size

The relatively small sample sizes of AD clinical studies also contributes to the challenge of reproducibility in AD integrative modeling. Many AD studies contain fewer than a thousand patients, and the longitudinal follow-up is limited. In addition, typically not all of the subjects were screened for the complete biomarker set, leading to sparse subsets of patients for whom the study contains complete data. As a result, models generated from these studies have a high margin of error and low statistical power, meaning they struggle to detect small effects.

The integration of different datasets into a larger dataset can overcome some of the challenges related to small sample sizes (Gomez-Cabrero et al., 2014). Integrated datasets provide more comprehensive data, and the resulting models have greater statistical power. However, current approaches for data integration were developed for the analysis of single-data-type datasets, and only subsequently adapted to handle datasets with multiple data types. For this reason, data integration methodologies can be ill-suited to manage the computational challenges arising from the variety of different data sizes, formats, and dimensionalities present in AD datasets, as well as their noisiness, complexity, and the level of agreement between datasets (Gomez-Cabrero et al., 2014; Gligorijević et al., 2015). Furthermore, even data acquired by analogous technologies are not necessarily integrable. For example, neuroimaging data acquired from similar scanners and similar modalities may still be stored in different formats and have different metadata content (Goble and Stevens, 2008).

Several strategies could be applied to address the interoperability challenges arising from data integration. The first strategy is to normalize and standardize data across all platforms (O'Bryant et al., 2015). However, scientific independency and freedom for innovation, as well as uniqueness of databases, must be respected. The second strategy is to collect a standardized set of biomarkers across different studies. Finally, the ideal solution would be performing a systematic longitudinal clinical and -omics follow-up of each individual in a large and rigorously characterized cohort since this would provide a statistically sufficient number of measurements in the context of subjects and variables. The Deep and Frequent Phenotyping study from Lawson et al. (2017) showed that such a cohort, in theory, is feasible. Yet, including a sufficient number of participants in such an ambitious study is costly.

#### Interpretability

In order for an AD model to have clinical impact, its findings must be interpretable. There are several barriers to AD model interpretability. Machine learning models often act as “black boxes”; it may be impossible to uncover the reasons for the predictions made by the model (Rudin, 2019). Indeed, as the number of features and the complexity of the computational processes used in models increases, this interpretability problem will worsen. Moreover, data-driven models are not causal and typically capture non-linear correlations between predictor and explanatory variables. While prior understanding of cause–effect relationships and detailed mechanisms might prove helpful to well-performing models, it is not necessarily required. Lack of mechanistic explanations for model prediction complicates the interpretation of data-driven findings and reduces acceptance by physicians (Fröhlich et al., 2018). Thus, the translation of data-driven models into a biomedical knowledge context is a major challenge in integrative AD modeling.

Combining available mechanistic knowledge with machine learning-based sub-models, so-called hybrid modeling could bridge the gap between experimental biological and computational research by improving interpretability (Fröhlich et al., 2018). For example, Bayesian networks which built on causal knowledge graphs constitute such a hybrid model (Arora et al., 2019). They shed light on interdependencies across features, which can be on different scales (e.g., clinical, genetic, and molecular), and allow for predicting the outcome of purely hypothetical clinical interventions. Similarly, other recent deep learning methodologies use knowledge-derived biological networks to define the layers of neural networks in order to improve interpretability (Fortelny and Bock, 2019).

## Conclusion

In the era of extensive biomarker profiling, big data, and artificial intelligence, integrative AD modeling comes with high promises. By integrating multi-scale, multimodal, and longitudinal patient data, such modeling approaches aim to provide a holistic picture of disease pathophysiology and progression. However, as we have discussed in this review, while integrative models have generated significant insights, and thus proved to be valuable in research, existing models do not yet fully describe critical aspects of AD.

The construction of hypothetical models simultaneously benefits and suffers from the vast amount of published knowledge. Prioritization of articles and computational text mining of literature corpora are reasonable approaches to identify a greater quantity of relevant knowledge while designing hypothetical models. In the field of data-driven integrative AD modeling, we highlighted several major ongoing challenges throughout the whole modeling process of data collection, integration of disparate data sources, data analysis, and model interpretation. Data missingness and data censoring are major bottlenecks in data collection as well as analysis and interpretation. Heterogeneity and complexity in biological data are major impediments to data integration, which is central to data-driven integrative modeling and validation. Data mapping, imprecise diagnostic stages, and biased data are barriers that hamper data analysis and interpretation. Furthermore, there is an insufficient number of subjects in studies, which restricts the statistical power of data-driven integrative AD models. Because of these challenges, to the best of our knowledge, at this point in time, there are no integrative AD models which have been used in clinical practice.

While in theory, certain existing integrative models are capable of predicting AD diagnosis and progression, they are not used in clinical practice. We see a number of steps that could bring us closer to the goal of precision medicine and that could enable patient diagnosis through integrative disease models in a clinical context. First, we, the AD research community, need to establish valid, informative biomarkers and clear criteria for AD diagnosis. This would result in reliable predictors that could be included in modeling approaches, as well as fewer diagnostic errors, which in turn reduce the effect of mislabeled data. Second, a global data schema that could support the normalization and standardization of data across measurements would ultimately facilitate improved data integration. If future cohort studies would adhere to such a schema, data integration would be straightforward and the cumulative time saved for researchers working with it would be enormous. Finally, innovative modeling approaches, such as causal inference techniques and hybrid modeling, which go beyond current state-of-the-art data-driven models by linking prior knowledge with data-driven models, need to be developed and made more robust. Overall, novel computational modeling approaches that surmount the current integrative AD modeling challenges may hold the potential to play an increasing role in the planning of medical interventions and practice.

## Author Contributions

SG drafted the manuscript. CR, CB, DD-F contributed to the final version of the manuscript. CH and MH-A reviewed the final version of the manuscript.

### Conflict of Interest

The authors declare that the research was conducted in the absence of any commercial or financial relationships that could be construed as a potential conflict of interest.

## Footnotes

^1^https://ourworldindata.org/causes-of-death

^2^https://www.thestreet.com/world/leading-causes-of-death-world-14869811

^3^https://www.alz.co.uk/research/statistics

^4^https://ourworldindata.org/causes-of-death

^5^https://library.ahima.org/doc?oid=65895#.Xdl-iZPYrOQ
